# With Thanks and Appreciation

**Published:** 2006-12-15

**Authors:** Eugene J Lengerich, James R Bohland, Pamela K Brown, Mark B Dignan, Brenda C Kluhsman, Electra D Paskett, Nancy E Schoenberg, Stephen W Wyatt

**Affiliations:** The Pennsylvania State University, Health Evaluation Sciences, Hershey, Pa; Virginia Polytechnic Institute and State University, Institute for Community Health, Blacksburg, Va; West Virginia University, Mary Babb Randolph Cancer Center, Morgantown, WVa; University of Kentucky, Markey Cancer Center, Lexington, Ky; The Pennsylvania State University, Health Evaluation Sciences, Hershey, Pa; The Ohio State University, Comprehensive Cancer Center, Columbus, Ohio; University of Kentucky, Markey Cancer Center, Lexington, Ky; University of Kentucky, College of Public Health, Lexington, Ky

## To the Editor:

On behalf of fellow contributors to the journal, we would like to thank you for choosing Appalachian health as the theme for the October 2006 issue of *Preventing Chronic Disease*. We appreciate your willingness to call attention to this persistent disparity in chronic disease, including cancer. This health disparity is unlike any other because it is defined by geography and interwoven with culture, socioeconomic position, and stereotypical image. In particular, we appreciate the professionalism your staff used in addressing this topic and in editing this issue throughout its full development.

We were particularly saddened to hear of the recent and sudden death of Barbara A. Mortimer, PhD, who worked tirelessly on the Appalachian issue. From our perspectives as authors and investigators, her professionalism made our job much easier. She not only added clarity to our writing but also helped bring much-needed attention to this important health disparity. She will be missed.

